# Purchase of ultra-processed foods in Norway: a repeated cross-sectional analysis of food sales in 2013 and 2019

**DOI:** 10.1017/S1368980023001192

**Published:** 2023-09

**Authors:** Marie Michaelsen Bjøntegaard, Marianne Molin, Marit Kolby, Liv Elin Torheim

**Affiliations:** 1 Department of Nursing and Health Promotion, Faculty of Health Sciences, Oslo Metropolitan University, P.O. Box 4 St. Olavs Plass, N-0130 Oslo, Norway; 2 Department of Nutrition, Faculty of Medicine, University of Oslo, Sognsvannsveien 9, 0372 Oslo, Norway; 3 Department of Health Sciences, Oslo New University College, NO-0456 Oslo, Norway

**Keywords:** Ultra-processed foods, NOVA classification, Food sales data, Norway, Dietary pattern, Food expenditure, Soft drinks

## Abstract

**Objective:**

A dietary pattern dominated by ultra-processed foods has been associated with non-communicable diseases in several studies. A previous study from 2013 found a high share of ultra-processed foods in Norwegian food sales. This study aimed to investigate the current share of ultra-processed foods in Norway and the development in expenditure on ultra-processed foods from 2013.

**Design:**

A repeated cross-sectional analysis of scanner data from the Consumer Price Index from September 2013 and 2019 and an investigation of the processing degree according to the NOVA classification system.

**Setting:**

Food sales in Norway.

**Participants:**

Norwegian grocery stores (*n* 180, for both time periods).

**Results:**

The share of expenditure in 2019 was highest for ultra-processed foods (46·5 %) and minimally or unprocessed foods (36·3 %), followed by processed foods (8·5 %) and processed culinary ingredients (1·3 %). An increasing degree of processing was found for several of the food groups between 2013 and 2019; however, most effect sizes were weak. In 2019, soft drinks became the most frequently purchased food item, surpassing milk and cheese, with the highest expenditure in Norwegian grocery stores. Increases in expenditure on ultra-processed foods were mainly due to increased expenditures on soft drinks, sweets and potato products.

**Conclusions:**

A high share of expenditure on ultra-processed food was found in Norway, which may imply a high consumption of these foods. The change in expenditure of NOVA groups between 2013 and 2019 was small. Carbonated and non-carbonated soft drinks were the most frequently purchased products in Norwegian grocery stores and contributed to most of the expenditures.

## Graphical abstract

**Figure:**
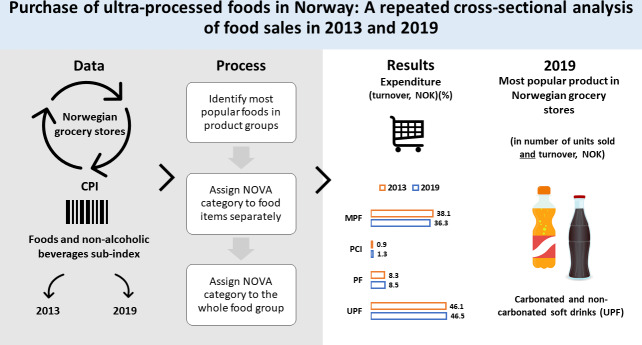

Traditional food processing, such as fermenting, cooking, baking, smoking, and salting foods, has been an integral part of culinary traditions throughout human evolution. Such processing has made foods safer, more digestible, increased the variety in flavours and allowed for the preservation of foods out of seasonal supply^(1)^. However, along with recent innovations in the food industry, there has been a large growth in industrially produced foods that are processed or modified to a greater extent than traditional products. The purpose of this is to create new and convenient foods, increase shelf life, change the nutritional composition or use cheaper ingredients. Such extensively processed foods were coined ‘ultra-processed’ by the introduction of the NOVA classification in 2009^(2)^. While the NOVA classification has been criticised for being ambiguous and difficult to interpret^(3)^, the researchers behind the classification system define ultra-processed foods (UPF) to be made by several steps of industrial processing that assemble food products from modified ingredients, such as derivatives of sugars, proteins, fats, starches, and fibres, with unmodified foods, and may include processes such as extrusion, moulding, and pre-frying^(4)^. UPF may also include additives used ‘cosmetically’ to enhance qualities such as colour, flavour, carbonation, foaming and thickening^(4)^.

The increase in both accessibility and intake of UPF has coincided with a global increase in obesity, CVD, cancer and other diseases known as non-communicable diseases (NCD)^(5–9)^. The association between a high intake of UPF and adverse health outcomes has partly been attributed to their high content of refined carbohydrates, added sugars, salts, saturated fats, and a lack of dietary fibre, which are dietary factors known to increase the risk of developing NCD^(10)^. Additional mechanisms by which UPF may negatively affect health include (1) changes made to the structure of foods, thereby affecting satiety signals mediated by the gut–brain axis, as well as biochemical changes of digested food components that affect the absorption of nutrients^(11)^, (2) the widespread inclusion of additives in food products, by which several of the most frequently applied additives may potentially cause negative health effects^(12)^ and the presently unknown ‘cocktail effect’ of consuming multiple additives concurrently since additives are individually risk assessed^(13)^, (3) the leaching of chemical substances through packaging materials affecting endocrine functions^(14)^ and (4) through induction of pro-inflammatory changes to the microbiome^(15)^. Additionally, (5) the experimental data on a UPF-dominated diet *v*. an ‘unprocessed’ diet imply that the inferior satiety potential of UPF can promote unintended overconsumption^(16)^, which could result in weight gain and thereby increase the risk of NCD.

National dietary guidelines in several countries now recommend reducing the consumption of UPF and increasing the intake of unprocessed or minimally processed foods (MPF), including in Brazil^(17)^ and Uruguay^(18)^, among others. While there is no specific recommendation to reduce the consumption of UPF in the Nordic countries, current Norwegian dietary guidelines recommend reducing the intake of several of the dietary components associated with these products, such as processed meats and foods with a high content of sugar and salt. Further, the preliminary conclusion from the Nordic Nutrition Recommendations 2022^(19)^ provides no recommendation to limit consumption UPF, as recommendations for other food groups sufficiently cover current evidence.

In Norway, data on individual intake levels of UPF are currently missing; however, the consumption of foods according to the NOVA classification can be estimated using food sales as a measure of consumption. Scanner data from the Consumer Price Index (CPI) provide nationally representative insight into food and beverage sales from Norwegian grocery stores. While data on food purchases cannot be extrapolated to households or individuals, these data provide a measure for population-level consumption and thus valuable information on consumption patterns in Norway. A previous study based on scanner data from the CPI in 2005 and 2013 found the share of expenditure on UPF to be 48·4 % and 48·8 %, respectively^(20)^.

This study aimed to investigate trends in consumption of UPF in the Norwegian population. To reach this objective, food sales data from September 2013 and 2019 were investigated, in terms of expenditure on NOVA groups, the most popular food items in-store, and geographical differences in Norway.

## Methods

### Research methods

The present study is a repeated cross-sectional analysis of secondary data from the CPI, in which food sales from two nationally representative samples of Norwegian grocery stores from September 2013 and September 2019 were analysed. The NOVA classification system^(4)^ was used to investigate the extent of food processing by assessment of ingredient lists on products made available by two online grocery stores.

### Description of data source

#### CPI

The CPI tracks historical developments in prices of Norwegian households’ goods and services. The main index consists of 12 sub-indices, including the ‘food and non-alcoholic beverage’ sub-index that was analysed in the present study. The CPI is connected to food sales data using identification codes in the barcode data of products in-store. The barcode data are linked to a consumer classification applied in the CPI, known as the Classification of Individual Consumption According to Purpose (COICOP)^(21)^, an international reference system of household expenditures^(22)^. The goal of this classification is to create homogenous categories of foods to be used in different types of statistical analysis. In 2016, the European Classification of Individual Consumption According to Purpose was launched. An overview of the food groupings can be found online^(23)^.

#### Population, sampling procedure and sample of grocery stores in the CPI

The target population includes grocery stores listed in Statistics Norway’s registry of businesses, coded 47·11 and 47·12. This includes most grocery stores on the Norwegian market and their associated kiosk outlets, given the ability to deliver electronic scanner data^(24)^. In 2013 and 2019, the population consisted of approximately 3899 and 3848 grocery stores, respectively, based on information from a market research company^(25)^. Grocery store chains include NorgesGruppen, Rema1000, Coop Norge, ICA, and Bunnpris.

The sampling procedure and data collection were completed by Statistics Norway^(26)^. Once a year, the pool of grocery stores that provide scanner data to the CPI is updated to ensure national representativity. The sampling procedure includes stratifying the study population based on the grocery store chain and store profile (budget, convenience, supermarket and kiosk). Then, based on the grocery stores’ turnover from sales, the Neyman allocation method was applied. Lastly, a selection of grocery stores was drawn randomly from the various strata by ‘simple random sampling without the possibility of replacement’.

There were approximately 180 grocery stores in the 2013 and 2019 datasets (Statistics Norway, personal correspondence, December 7th, 2020). Narvesen was the only kiosk outlet represented and constituted a minor part of the final sample (Statistics Norway, personal correspondence, November 18th, 2020). Specialty stores, gas stations and cross-border trade were not included^(26)^. Statistics Norway has not released information on the final sample characteristics to ensure the anonymity of the included grocery stores. However, since the sampling procedure was based on turnover from sales, market shares from 2013 and 2019 can provide insight into the distribution of grocery store chains in the sample. In 2013 and 2019, NorgesGruppen contributed to 39 % and 44 % of Norwegian food retailers, followed by Rema1000 (23 %-share in both years), Coop (23 % and 29 %, respectively) and Bunnpris (4 %)^(25,27)^. ICA held 11 % of the market share in 2013 but was acquired by Coop in 2014.

#### Data collection in the CPI

In the CPI, data from the sample of grocery stores are collected electronically through an automated process^(26)^. A product sale in-store generates electronic data with information about the sale and is subsequently registered at the grocery store’s main office. Once a month, price summaries for all product sales are forwarded to Statistics Norway for inclusion in the CPI.

### Data variables

A total of 501 938 price observations were registered in September 2013 and 662 957 in September 2019. Each price observation represented the total sales of a specific product in a particular grocery store. Data were obtained on category level (i.e. milk) rather than product level to avoid the inclusion of datasets with a high amount of missing information, caused by the extensive anonymisation of brands in the original datasets.

The COICOP groups were organised into the most detailed level possible, resulting in 107 and 108 separate product groups (i.e. apples, chicken) in 2013 and 2019, respectively. To compare between different food groups, we aggregated the 109 product groups into the 12 food groups (i.e. vegetables, fruits and dairy, meat and poultry) from the annual report Developments in the Norwegian Diet^(28)^. Lastly, the price observations included the county code as point of sale. These were grouped into geographical areas in Norway, namely North, South, East, West, Central, and City of Oslo, based on county codes in 2013 and renewed country codes in 2019 (Table 1).

**Table 1:** Geographical regions in Norway organised by county codes in 2013 and renewed county codes in 2019

Geographical areas in Norway	2013 County name (code)	2019 County name (code)
South	Aust-Agder (09), Vest-Agder (10)	Agder (42)
North	Nordland (18),Troms (19), Finnmark (20)	Nordland (18), Troms og Finnmark (54)
Central	Sør-Trøndelag (16), Nord-Trøndelag (17)	Trøndelag (50)
East	Østfold (01), Akershus (02), Buskerud (06), Hedmark (04), Oppland (05), Vestfold (07), Telemark (08)	Viken (30), Innlandet (34), Vestfold og Telemark (38)
West	Rogaland (11), Hordaland (12), Sogn og Fjordane (14), Møre og Romsdal (15)	Rogaland (11), Vestland (46), Møre og Romsdal (15)
City of Oslo	Oslo (03)	Oslo (03)

### Food classification

The NOVA classification system was used to categorise food products according to processing degree. An in-depth description and examples of the NOVA groups can be found in online supplementary material, Supplemental Table 1 in the electronic article. The framework includes four groups that describe the purpose and extent of food processing^(4)^: group 1 (unprocessed or minimally processed foods), group 2 (processed culinary ingredients), group 3 (processed foods) and group 4 (ultra-processed foods).

For each product group, we identified the most frequently sold food items and based the classification on these. Sorting food items by frequency of purchase was an available feature of the online grocery stores of Meny and Spar, by using the filter function. The search function also made ingredient lists available for evaluation to aid the classification. A product group was categorised as a NOVA group if > 75 % of food items in the product group were classified as such. A food item refers to a specific product investigated. An additional group (NOVA-X) was created for unclassifiable product groups, in which < 75 % of food items were classified into a single NOVA group. In total, 107 product groups in 2013 and 108 product groups in the 2019 were categorised according to the NOVA classification guidelines. To increase the reliability and validity of our findings, efforts were made to systemise the process of classifying the products. This included the development of a clear procedure that was applied during the NOVA classification process, and additionally, a checklist based on the NOVA guidelines^(4)^ (Table 2 and see online supplementary material, Supplemental Table 2, respectively). The checklist was followed from start to finish for each product.

**Table 2:** Procedure for classifying foods, developed for the present study to guide the NOVA categorisation of food products

**PROCEDURE FOR CLASSIFYING FOODS**		
1	Online search and identification of the most popular products	
	▪	Online search: Meny and Spar online grocery stores; filter function to sort products from the most to the least frequently purchased; number of products recorded in Microsoft Excel
	▪	Excel automatically calculates the number of products to investigate*
2	Investigate products individually and assign NOVA group	
	▪	Inspect the ingredient lists of all products selected in Step 1 and follow the checklist
	▪	Record the NOVA categories given to all products in Excel
3	Assign NOVA category to the whole product group	
	▪	The overall NOVA group is automatically assigned using the percentage shares and the COUNTIFS formula in Excel†
**NOVA categorisation is finalised**		

*All products were investigated in the small product groups (< 10 products), 2/3 of products were investigated in the medium-sized product groups (> 10–29 products), and 1/3 of products were investigated in the large product groups (> 30 products), to reflect that products in the smaller product groups had a higher likelihood of being purchased than products in the larger product groups.

^†^To assign an overall NOVA category to a product group (i.e. canned fish), 75 % of the individual products within the product group needed to belong to a single NOVA group. If 75 % of the products did not belong to a single NOVA group, the product group was deemed to be unclassifiable.

### Data analysis

The turnover of expenditure was used as an indicator of food consumption. The turnover share was calculated using Pivot Tables from Microsoft Excel and was calculated as the percentage share of the total turnover from sales (NOK). In investigation of the sales of different product groups, the share of purchases in terms of numbers of units sold (litres, kilograms, singlepacks or multipacks) was also studied. Pivot Tables aggregated food sales data into tables in Excel. The absolute difference in share of expenditure between 2013 and 2019 was calculated in Excel, by subtracting the %-share in 2013 from the %-share in 2019.

Statistical analysis was performed in R version 4.0.4. The Pearson chi-square test for independence was used to test for statistically significant change in variables between 2013 and 2019. Due to the high count of frequencies in the sample, the chi-square test was extremely sensitive and all *P*-values were very low (*P* < 0·001), displaying false-positive associations. A Bonferroni correction of the *P*-values did not alter results. Since the effect size is not affected by the high count of frequencies in the sample, a Cramer’s V-test was completed to determine the effect size of the *X* ^2^. Due to the false-positive chi-square tests, the *P*-values are not presented in the present article and only the Cramer’s V-test was used to assess the strength of the association. A Cramer’s V value between 0 and 1 is given, in which a test result > 0·25 demonstrates a very strong relationship, 0·15–0·24 = strong, 0·10–0·14 = moderate, 0·05–0·09 = weak and < 0·05 finds no or a negligible association between the two variables^(29)^. The chi-square test and Cramer’s V were performed on NOVA groups 1–4 only, due to the ordinal nature of the NOVA groups.

## Results

### Change in expenditure on NOVA groups

**Fig. 1:**
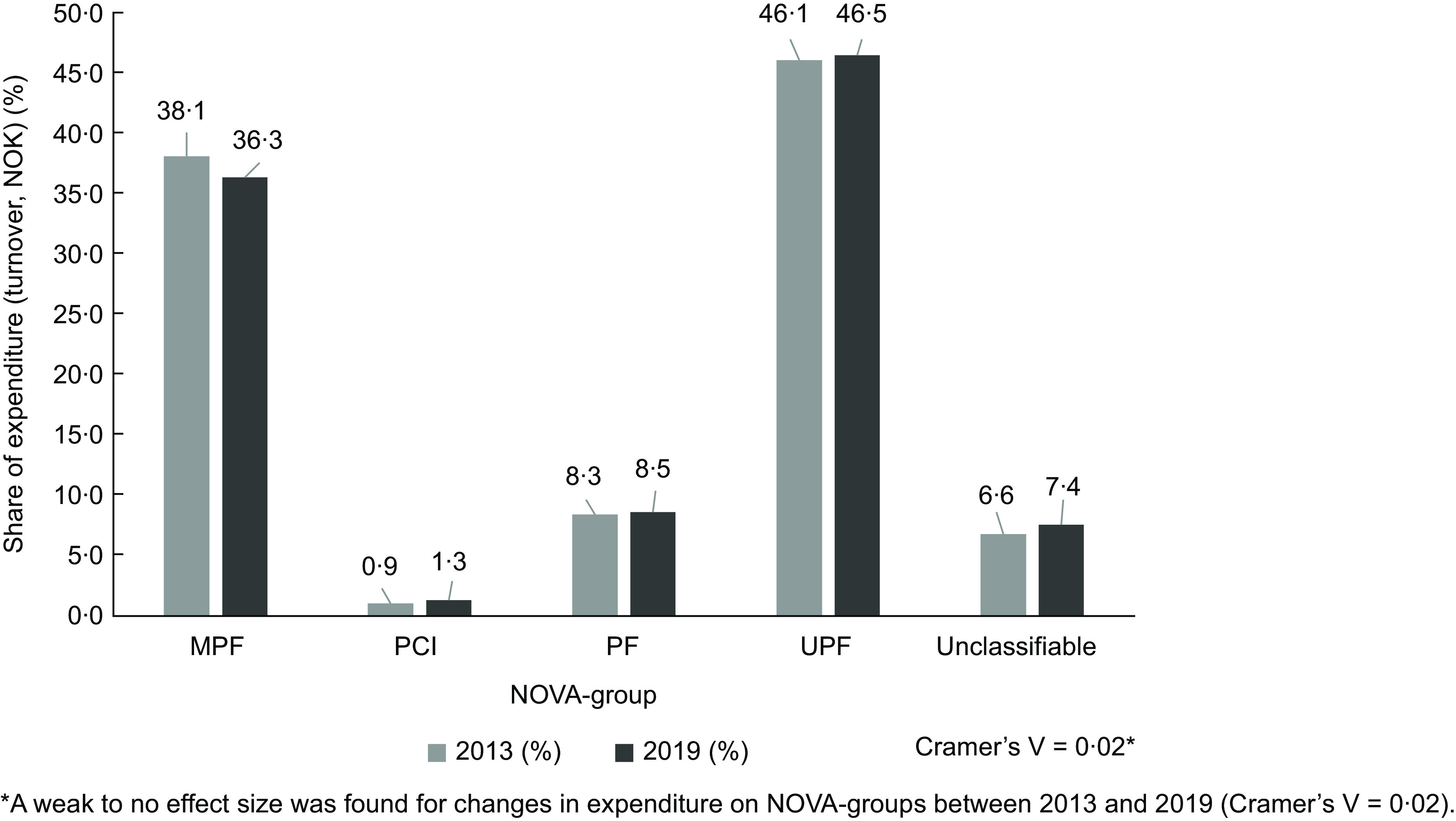
The distribution in the share of expenditure (turnover, NOK) on NOVA groups (NOVA groups: MPF, minimally or unprocessed foods; PCI, processed culinary ingredients; PF, processed foods; UPF, ultra-processed food) in 2013 and 2019. *A weak to no effect size was found for changes in expenditure on NOVA groups between 2013 and 2019 (Cramer’s V = 0·02).

Firstly, the shares of expenditure on NOVA groups in 2013 and 2019 were investigated (Fig. 1). The total expenditure was 596 million NOK in September 2019, in which 46·5 % of expenditure was ascribed to UPF and 36·3 % to MPF. There were only small shares of expenditures on processed culinary ingredients (PCI) (group 2) and PF (group 3). The unclassifiable group contributed to a relatively large share of expenditures with 7·4 %. Overall, there was no noteworthy difference in the distribution of expenditure on NOVA groups between 2013 and 2019 (Cramer’s V = 0·02).

### Change in expenditure on NOVA groups in the food groups between 2013 and 2019

**Fig. 2:**
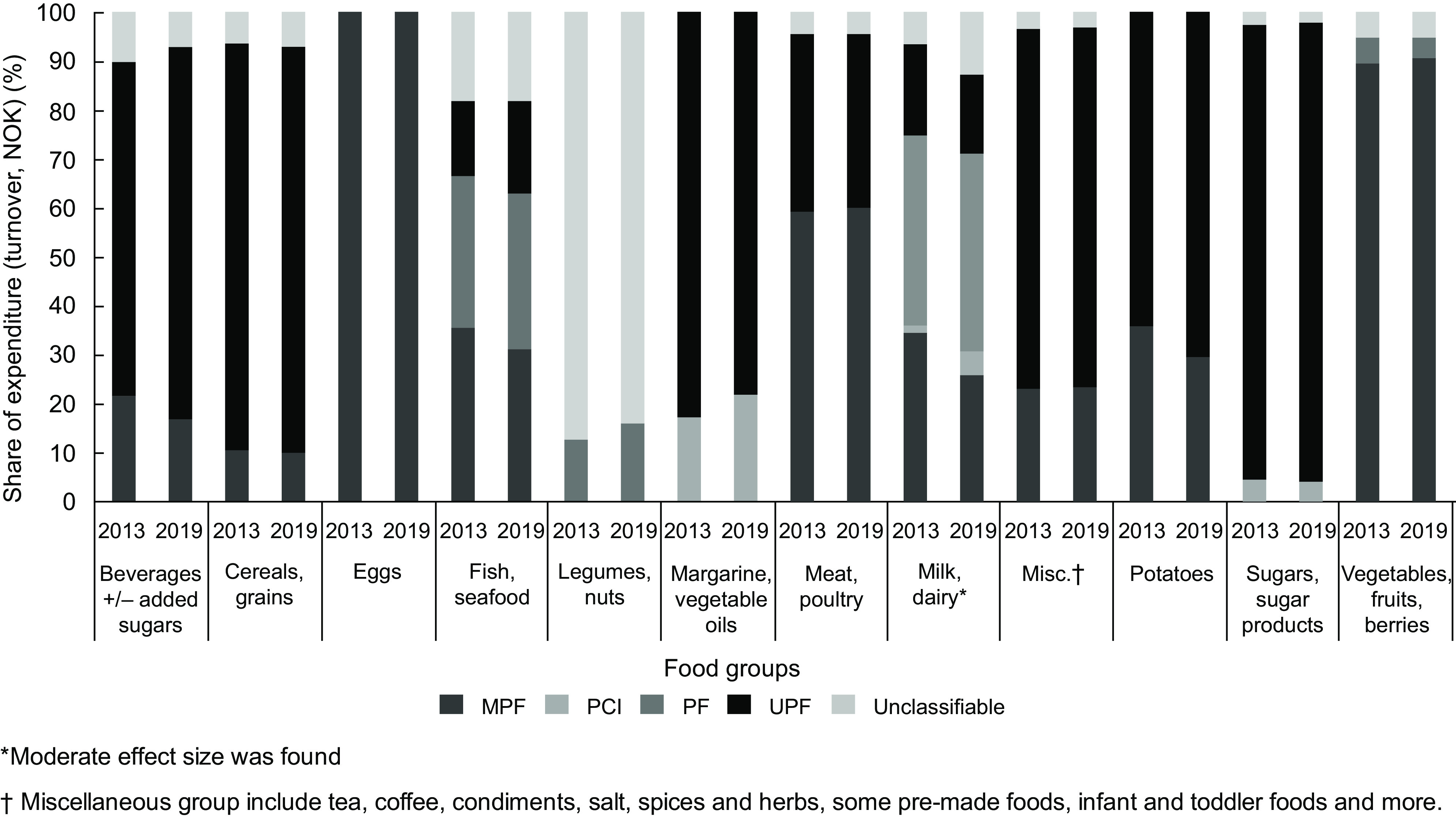
Distribution of the share of expenditure (turnover, NOK) (%) on NOVA groups (NOVA groups: MPF, minimally processed foods; PCI, processed culinary ingredients; PF, processed food; UPF, ultra-processed food) in the food groups (*n* 12) in 2013 and 2019. *Moderate effect size was found. †Miscellaneous group include tea, coffee, condiments, salt, spices and herbs, some pre-made foods, infant and toddler foods and more.

There were some changes in the expenditure on NOVA groups in the food groups (*n* 12) in the time period; however, most of the effect sizes were weak. A complete overview of the results can be found in Fig. 2 and online supplementary material, Supplemental Table 3. For *Beverages with or without sugars,* expenditure on UPF increased by 7·8 % due to increased sales of carbonated and non-carbonated soft drinks, specifically. Decreases of 4·8 % and 3·0 % in expenditure were found for MPF (nectars and juice) and the unclassifiable group (mineral water, excluding soft drinks), respectively. For *Fish and seafood,* expenditure increased 3·9 % for ultra-processed fried and breaded fish products, whereas it decreased 4·4 % for minimally processed fish. For *Margarine and vegetable oils*, expenditure on UPF (margarine) was reduced by 4·6 % and increased by the same share for PCI (vegetable oils). The effect sizes were found to be weak (Cramer’s V < 0·06). For ultra-processed *Cereals and grains*, expenditure increased for some products, that is, 3·4 % and 2·5 % increased expenditure on ready-to-bake cake mixes and cakes and decreased for other products, such as 6·7 % and 3·3 % reduced expenditures on bread and cookies, respectively. However, the effect size was negligible for this group (Cramer’s V = 0·005). For *Milk and dairy*, expenditure on MPF (milk) decreased 8·6 % between 2013 and 2019, and the expenditure increased 3·2 % on PCI (butter) and 1·7 % on PF (cheese). Surprisingly, the overall expenditure on ultra-processed milk and dairy products decreased 2·4 %. However, the expenditure increased 6·1 % on the unclassifiable group *Assorted dairy products* that was introduced in the 2019 dataset, which could explain part of the reduced expenditure observed on ultra-processed milk and dairy products. A moderate effect size was found for changes observed for *Milk and dairy* (Cramer’s V = 0·10). The *Miscellaneous* group comprised mostly of a variety of UPF, but also some beverages (tea, coffee) and salt, spices and herbs. A 7·4 % increase was found in expenditure on dinner bases and kits and 4·2 % increase on pre-made dinners. The expenditure decreased 3·9 % on sauce and gravy and by 3·8 % on ready-made salads. Effect sizes were negligible (Cramer’s V < 0·01). In the *Potato* group, expenditure increased 6·3 % on UPF (potato products and chips), whereas it decreased by 6·3 % on fresh and minimally processed potatoes (Cramer’s V = 0·05, weak effect size).

### Investigation of the most popular foods and beverages

We investigated the most popular foods and beverages, represented by product groups (*n* 108) with the highest expenditure and the highest numbers of units sold, in September 2013 and 2019. The ten product groups with the highest expenditure and the most units sold in September 2013 and 2019 are presented in Table 3 (full overview in online supplementary material, Supplemental Tables 4 and 5, respectively). In 2019, soft drinks were the product group most frequently bought and comprising the highest expenditure. Other popular UPF included breads, chocolates and pre-made dinners in terms of expenditure, and breads, yoghurts, baguettes and bread rolls, and chips in terms of number of purchases. Many popular foods were classified as MPF, including expenditures on different types of meats, poultry, milk, fresh vegetables and purchases of milk, fresh vegetables and stone fruits. Additionally, for PF, cheese contributed to high shares of expenditures and purchases.

**Table 3:** The ten most popular product groups in terms of share of purchase (a) and expenditure (b) in 2013 and 2019

A) Purchase (units sold)				A) Purchase (units sold)			
#	Product group	NOVA*	2013 (%)	#	Product group	NOVA	2019 (%)
1	Milk	1	6·6	1	Carbonated/ non-carbonated soft drinks	4	5·9
2	Carbonated/ non-carbonated soft drinks	4	5·6	2	Milk	1	4·4
3	Bread	4	4·5	3	Cheese	3	4·0
4	Cheese	3	3·6	4	Bread	4	3·5
5	Yoghurt	4	3·5	5	Yoghurt	4	3·4
6	Chocolate	4	3·4	6	Chocolate	4	2·8
7	Baguettes and bread rolls	4	2·6	7	Other fresh vegetables	1	2·5
8	Other fresh vegetables	1	2·6	8	Baguettes and bread rolls	4	2·4
9	Stone fruits	1	2·3	9	Chips†	4	2·3
10	Minced meat, meatballs, and meat patties	1	2·1	10	Stone fruits	1	2·1

B) Expenditure (turnover, NOK)				B) Expenditure (turnover, NOK)			
#	Product group	NOVA*	2013 (%)	#	Product group	NOVA	2019 (%)
1	Cheese	3	5·8	1	Carbonated/ non-carbonated soft drinks	4	6·3
2	Carbonated/ non-carbonated soft drinks	4	5·4	2	Cheese	3	6·0
3	Milk	1	4·5	3	Bread	4	3·7
4	Bread	4	4·3	4	Minced meat, meatballs, and meat patties	1	3·3
5	Minced meat, meatballs, and meat patties	1	3·6	5	Milk	1	3·3
6	Chocolate	4	2·9	6	Chocolate	4	2·7
7	Poultry	1	2·5	7	Other fresh vegetables	1	2·3
8	Yoghurt	4	2·4	8	Poultry	1	2·3
9	Cold cuts	4	2·2	9	Mutton, lamb, and goat	1	2·2
10	Stone fruits	1	2·1	10	Pre-made dinners	4	2·1

*NOVA groups: 1, minimally or unprocessed foods; 2, processed culinary ingredients; 3, processed foods; 4, ultra-processed foods.

^†^Split from potato products in the 2019 dataset.

### Geographical differences in expenditure on NOVA groups

Regional differences in the expenditure on NOVA groups in Norwegian grocery stores between 2013 and 2019 were also investigated (see online supplementary material, Supplemental Table 6). Overall, the shares of expenditures on NOVA groups across Norway were similar. Expenditures on UPF ranged from 45·3 % in Oslo and in the North of Norway to 47·4 % in Central Norway. Between 2013 and 2019, expenditure on UPF decreased in the South and in the North of Norway, but increased in Central, West and East of Norway, and in Oslo. The changes were weak (Cramer’s V < 0·07) for most of Norway and very weak (Cramer’s V < 0·05) for Central and North of Norway. For MPF, the shares of expenditures ranged from 34·9 % in Central Norway to 37·9 % in the North of Norway. Decreased expenditures were found in all of Norway, and the largest decline of 4·5 % was found in Oslo. The effect sizes were weak (Cramer’s V < 0·07) for East, West and South of Norway, and negligible for Central Norway, the North and the city of Oslo (Cramer’s V < 0·05). Interestingly, Central Norway had both the highest share of expenditure on UPF and the lowest expenditure on MPF, whereas the opposite was found in the North of Norway. For PF, expenditure ranged from 8·0 % in the West of Norway to 9·5 % in the South. Between 2013 and 2019, there were small increases in expenditure on PF in most of Norway (Cramer’s V < 0·05, negligible effect size), apart from a decrease as observed for Oslo (Cramer’s V = 0·11, moderate effect size) and in the West of Norway (Cramer’s V = 0·03, negligible effect size). For PCI, a slight increase was found between 2013 and 2019, and the shares of expenditures ranged from 1·2 % in the West of Norway to 1·4 % in Central Norway and in Oslo. The changes observed for PCI were strong (Cramer’s V < 0·23) for the East, West, North and Central Norway, very strong (Cramer’s V = 0·25) for the South and weak (Cramer’s V = 0·05) for Oslo.

## Discussion

The purpose of this study was to investigate the consumption of ultra-processed foods (UPF) in Norway by analysing scanner data in a nationally representative sample of grocery stores (*n* ≈ 180) in September 2013 and 2019. This study found that most of expenditure were attributed to either UPF (46·5 %) and minimally or unprocessed foods (MPF) (36·3 %) for both time periods. Our investigation of changes in the expenditure on food groups found a shift from MPF to UPF for some food groups. However, most of the effect sizes were weak or negligible, except for the moderate effect size observed in the *Milk and dairy* group. In 2019, soft drinks were the top expenditure product group in Norwegian grocery stores, surpassing milk. Additionally, our study found that increases in expenditure on UPF in most of Norway were mainly due to increased expenditures on soft drinks, sweets and potato products.

### UPF constitute most of expenditures in Norwegian grocery stores

Overall, the present study found a high share of expenditure on UPF. While the expenditure on MPF decreased between 2013 and 2019, there was a slight increase in the expenditure on UPF. However, the changes were small; from 46·1 % in 2013 to 46·5 % in 2019.

A few studies have measured the consumption of UPF through food sales. A previous study investigated expenditure (turnover from sales, NOK) on UPF in Norwegian retailers in September 2005 and 2013^(20)^. Overall, our results from 2013 compare well. Similarly to the present study, the authors found a slight increase in the share of expenditure on UPF between 2005 and 2013, from 48·4 % to 48·8 %. MPF also contributed to a large share of food sales, with 31·8 % and 33·0 % of food sales in 2005 and 2013, respectively. Expenditures on PCI and PF were somewhat higher than our results, and the shares were reduced between 2005 and 2013^(20)^, whereas it increased in our study. Since both our results from 2013 are from September, the differences in results would be of methodological nature. For instance, the lower share of PF found in the present study may be attributed to canned fish foods being unclassifiable in our study and classified as processed in the previous investigation^(20)^.

### Share of UPF in the diet, dietary quality, and health

In our findings, the region with the highest share of expenditure on UPF also had the lowest share of expenditure on MPF. This is in line with other studies suggesting that UPF may displace foods of a lower degree of processing^(30)^. A high consumption of UPF has been associated with an inferior nutritional quality in several countries, including France^(31)^, Belgium^(32)^ and the USA^(33)^, among others. Epidemiological studies have found a high consumption of UPF to be associated with increased consumption of dietary energy, carbohydrates, added sugars, sodium and saturated fats, and less proteins, fibre and several vitamins and minerals. A high consumption of UPF has been positively associated with obesity and NCD, such as diabetes, irritable bowel syndrome, heart disease, some types of cancers and all-cause mortality^(7–9,34)^.

In Norway, NCD are a major public health threat, representing the main cause of death and disease^(35)^. NCD also constitute a high economic cost for the Norwegian society due to lost productivity and treatment of diseases^(36)^. Hence, a reduced consumption of UPF may reduce the public health burden of NCD in Norway. Measures to shift consumption towards a higher share of whole and minimally processed foods at the expense of UPF could be a part of governmental food and nutrition strategy. However, due to the complexity of classifying UPF, this should not be left to the individual consumer but should rather be a responsibility of the government.

In 2017, the Global burden of Disease study investigated the role of an inferior diet and the risk of developing NCD^(37)^. Of the 11 million deaths and 255 million DALYs attributable to dietary risk factors, a high intake of sodium and low intake of whole grains and fruits were the leading risk factors globally. Whole grains contribute to the consumption of dietary fibres, which have a *convincing* protective effect against colorectal cancer^(38)^. Meanwhile, Norway rank high on the global prevalence of colorectal cancer^(39)^ and consumption of dietary fibre in 2010–2011 was found to be below the recommendations^(40)^. Store-bought bread is an important source of whole grains and dietary fibre in the Norwegian population and a staple food. Bread from Norwegian grocery stores was classified as UPF in the present study and contributed to a large share of UPF sales. A reduced consumption is therefore recommended as per the NOVA classification. This may not be relevant elsewhere if store-bought breads classify as group 3. However, in Norway, most of retail breads were ultra-processed and since baking bread at home may be unfeasible, a reduced consumption of store-bought bread may decrease the consumption of whole grains if no other dietary source is included. A recent systematic review and meta-analysis found ultra-processed whole grain cereals to be significantly and negatively associated with risk of all-cause mortality^(34)^. Thus, caution should be taken to prevent reducing the consumption of nutrients that are known to be protective of certain diseases, such as dietary fibre.

### Less expenditure on milk and more on dairy products and soft drinks

A moderate effect size (Cramer’s V = 0·10–0·15) was found for changes in expenditure on NOVA groups in the *Milk and dairy* food group between 2013 and 2019, mainly due to a large reduction in expenditure on milk and an increase in expenditure on assorted dairy products (unclassifiable group). In 2005, the share of expenditure on milk was 5 %^(20)^, and in the current study, the expenditure decreased from 4 % to 3 % between 2013 and 2019.

Some of the reduced expenditure on milk may be explained by the introduction of a new product group in the 2019 dataset, namely *Assorted dairy products*. This product group was unclassifiable due to the variety of included products ranging from cultured milk to flavoured milk and protein puddings. However, the group consisted of 67 % UPF and thereby close to being classified as such. In general, dairy foods contributed to a relatively large share of food sales, and an increase in expenditure between 2013 and 2019 was found for dairy-based desserts and snacks. Similar findings have been reported in a global analysis of food sales, in which dairy products were found to be the second most important contributing food group to UPF^(41)^. It is plausible that extensively processed dairy products contribute to a high and increasing share of UPF consumption in Norway; however, due to the restructuring of COICOP groups between 2013 and 2019, we could not accurately examine this. Increased popularity of plant-based alternatives may have led to reduced expenditure on dairy-based milk. However, in the present analysis, the structural nature of COICOP groups included the sales of plant-based alternatives in the sales of dairy-based milk, and this could not be further examined.

Soft drinks became the most popular food item in Norwegian grocery stores in 2019, both in terms of shares of expenditure and in the number of units sold. The present study was unable to distinguish between artificially sweetened and sugar-sweetened beverages; however, a large increase in consumption of artificially sweetened beverages, specifically, was recently found in Norway^(28)^. Our investigation did not include food sales in venues other than grocery stores, such as cross-border trade, kiosks (besides Narvesen), gas stations, restaurants and specialty stores, where soft drinks may be frequently purchased. The consumption of soft drinks was therefore likely underestimated in the present analysis. For instance, between 2010 and 2019, expenditure on soft drinks and mineral water in cross-border trade (excluding cafes, restaurants and services) increased from 5·6 % to 10·6 % of total expenditure^(28)^.

### Application of the NOVA classification system

The NOVA classification is the most widely applied and recognised framework to assess the degree of processing of foods in the literature, and many studies have consistently found a positive association between UPF and adverse health outcomes^(6–9)^. Additionally, the NOVA classification system was recently validated for identifying UPF using food processing biomarkers^(42)^. Still, the classification system has been criticised for disregarding the role of food texture rather than processing degree in increasing energy intake^(43)^, though these two concepts may be viewed as highly correlated. Further, a common limitation of many investigations, including the present study, is using survey methods not designed and validated to assess the degree of processing of foods^(44)^. The classification system has also been criticised for being difficult to interpret and demonstrated a low consistency in categorising NOVA groups among food and nutrition specialists^(3)^.

An increasing level of detail is found in household budget surveys and dietary registration of individuals, which describe differences in consumption within the population or by individuals. Food sales data could be more comparable with these methods by using data on product level. This would decrease misclassification, by allowing unclassifiable product groups, such as Mineral water containing unflavoured water (group 1) and flavoured water (group 4), to be disaggregated and classified accordingly. A greater level of detail in the data would also reduce the difficulty in comparing consumption trends due to restructuring of COICOP groups.

The guidelines provided a detailed description of UPF. However, knowledge of additives, ingredients and processing techniques is required to classify products according to the NOVA classification. Without this knowledge, the risk of misclassification is considerable. Efforts have been made to reduce misclassification bias in the present study, by using checklists, applying a systemised process, basing the categorisation on the most popular products only and consulting with experts. However, some misclassification was unavoidable.

### Strengths and limitations

The current study investigated expenditures in grocery stores and a small number of kiosks. Most food purchases in Norway, apart from alcoholic beverages, are made in grocery stores rather than specialty stores and cross-border trade^(45)^, and our results are therefore representative of food purchases in Norwegian grocery stores. Purchases in restaurants contribute to a fair share of food purchases^(45)^, but were not investigated in the present study.

In between purchasing foods and consuming foods, many factors may play a role and affect the actual food intake, such as food waste, preparation and distribution among family members. Therefore, food sales data can only indirectly examine food consumption^(46)^ and our findings should be interpreted with caution.

This study is a population-level investigation of the Norwegian diet and does not represent consumption of UPF in individuals or households. However, the data allow for comparison of food trends over time which can provide valuable insights into changes in Norwegian dietary patterns. Also, another advantage of using food sales data in nutrition research is the objectivity of the data, avoiding recall bias and potential misreporting, which are known limitations to other methods that collect food intake data^(47)^.

The present study did not include alcoholic beverages. The NOVA classification proposes that fermentation of group 1 foods, such as beers and ciders, classifies as group 3 foods^(4)^. No data was found for sale of alcoholic beverages in the retail sector alone; however, combined with sales in licensed venues, beers contributed to most of the sales in 2018^(48)^. Consequently, caution should be made when comparing results with other studies that include alcoholic beverages.

In the investigation of the most popular foods, both shares of expenditures and purchases were used in the analysis. While both shares of expenditures and purchases can be used as indirect indicators for consumption, they offer slightly different information. Expenditure describes the value associated with the product and our willingness to spend. Purchases are associated with frequency, in which a food item that is purchased more may indicate a habitual purchase, whereas fewer purchases may represent foods consumed more rarely. This information can be valuable for research on consumer behaviour and to find entry points for interventions on a public health level.

## Conclusion

This study investigated the degree of processing of Norwegian food sales and found a high share of expenditure on UPF, indicating a high consumption of these foods. Between 2013 and 2019, changes in expenditure were small both when considering NOVA groups and geographical differences in Norway. Carbonated and non-carbonated soft drinks became the most popular food item regarding both expenditure and purchase in Norwegian grocery stores, surpassing milk and cheese.

## Supplementary material

For supplementary material accompanying this paper visit https://doi.org/10.1017/S1368980023001192. click here to view supplementary material
